# The Association between Non-Invasive Hepatic Fibrosis Markers and Cardiometabolic Risk Factors in the Framingham Heart Study

**DOI:** 10.1371/journal.pone.0157517

**Published:** 2016-06-24

**Authors:** Michelle T. Long, Alison Pedley, Joseph M. Massaro, Udo Hoffmann, Caroline S. Fox

**Affiliations:** 1 Division of Gastroenterology, Boston Medical Center, Boston University School of Medicine, Boston, Massachusetts, United States of America; 2 National Heart, Lung, and Blood Institute’s Framingham Heart Study, Framingham, Massachusetts, United States of America; 3 Merck Research Laboratories, Kenilworth, New Jersey, United States of America; 4 Department of Mathematics and Statistics, Boston University, Boston, Massachusetts, United States of America; 5 Radiology Department, Massachusetts General Hospital, Harvard Medical School, Boston, Massachusetts, United States of America; 6 Division of Endocrinology, Hypertension, and Metabolism, Brigham and Women’s Hospital, Harvard Medical School, Boston, Massachusetts, United States of America; RWTH Aachen, GERMANY

## Abstract

**Background & Aims:**

Non-alcoholic fatty liver disease (NAFLD) is associated with an increased risk of cardiovascular related death, particularly in those with hepatic fibrosis. We determined the prevalence of predicted fibrosis based on non-invasive fibrosis markers and the association of hepatic fibrosis with cardiovascular risk factors.

**Methods:**

Cross-sectional study of 575 Framingham Heart Study participants with NAFLD based on computed tomography. We determined the prevalence of predicted fibrosis based on the aspartate aminotransferase (AST)/alanine aminotransferase (ALT) ratio, AST to platelet ratio index (APRI), the Fibrosis-4 score (FIB4), and the NAFLD Fibrosis Score (NFS). Using multivariable logistic regression models, we examined the association between low, indeterminate, or high risk for fibrosis according to the NFS and various cardiometabolic risk factors.

**Results:**

The predicted risk of fibrosis was 12%, 4%, 5%, and 32% for the NFS, FIB4, APRI, and AST/ALT ratio, respectively. In multivariable models, participants with a high risk for advanced fibrosis by the NFS had a wider pulse pressure (adjusted mean difference = 6.87 mm Hg; p = 0.0002) and an increased odds of hypertension (OR 2.92; p = 0.007) compared to those with low risk of fibrosis. There were no statistically significant differences between other cardiovascular risk factors for those with a high versus low risk of fibrosis.

**Conclusions:**

The AST/ALT ratio, APRI, and NFS give widely disparate predictions of liver fibrosis. Participants with a high risk for fibrosis based on NFS had wider pulse pressure and increased odds of hypertension. Whether modifying these risk factors impacts cardiovascular endpoints in NAFLD patients remains unknown.

## Introduction

Non-alcoholic fatty liver disease (NAFLD) affects approximately 30% of the United States population and is considered the most common chronic liver condition [1, 2]. NAFLD encompasses a spectrum of liver pathology including simple steatosis, non-alcoholic steatohepatitis (NASH) with varying amounts of fibrosis, and cirrhosis. While NAFLD is common, the prevalence of NASH is lower at about 5–10% of the US adult population [2].

There are several challenges in studying NASH on the population level. First, the gold standard for diagnosing NASH is a liver biopsy which is costly, burdensome, and not practical to implement on a large scale [3]. Second, traditional imaging techniques including ultrasound, computed tomography (CT) scan, and magnetic resonance imaging are insensitive to the pathological features of NASH, including liver inflammation and fibrosis [4]. Additionally, as the liver becomes more fibrotic, steatosis diminishes which may make the diagnosis difficult [5]. To combat these challenges, several non-invasive tools have been developed to diagnose hepatic fibrosis, including serum biomarker panels [6]. Available serum based tests include the simple aspartate aminotransferase (AST) to alanine aminotransferase (ALT) ratio [7], the AST to platelet ratio index (APRI) [8], and the Fibrosis-4 score (FIB4)[9] to the more complex NAFLD fibrosis score (NFS) [10]. These markers have been validated to identify and exclude advanced fibrosis in patients with NAFLD [6, 11, 12]. Currently, there is a lack of consensus on the optimal surrogate marker of hepatic fibrosis to be used in population-based studies [3, 6].

NAFLD patients with evidence of fibrosis as predicted by non-invasive fibrosis markers have an increased risk of liver- and cardiovascular-related complications and death [13, 14]. It is not known if the non-invasive fibrosis markers are predicting increased cardiovascular death because of worsened liver fibrosis or because of concurrent cardiovascular disease. The relationship between liver fibrosis and traditional cardiometabolic risk factors in the general population is not known and may help inform future studies that assess liver fibrosis as an independent risk factor for cardiovascular mortality.

Thus, we aimed to determine the prevalence of predicted hepatic fibrosis using the non-invasive markers AST/ALT ratio, APRI, FIB4, and NFS in a community-based cohort study. Additionally, we determined the association of hepatic fibrosis with an adverse cardiometabolic profile.

## Materials and Methods

### Study sample

The Framingham Heart Study (FHS) is a multi-generational, community based cohort study which has previously been described in detail [15]. For the present investigation, our sample was derived from a total of 2,803 Offspring, Third Generation, and Omni 2 Cohort participants who also participated in the multi-detector CT 2 sub-study for evaluation of ectopic fat, including liver fat, between September 2008 and December 2011. Since we were primarily interested in non-alcoholic fatty liver disease, we excluded participants with significant alcohol use (n = 425) defined as > 7 drinks per week for women and > 14 drinks per week for men. We additionally excluded anyone with missing covariate information or missing components of the non-invasive fibrosis markers (n = 410), yielding a total sample of 1,968 participants (962 Offspring, 138 Omni, and 868 Third Generation). The institutional review boards of the Boston University Medical Center and Massachusetts General Hospital approved the study protocol. All participants provided written informed consent.

### Measuring fatty liver and visceral adipose tissue

The Multi-detector CT scan protocol has been described in detail previously [16, 17]. A calibration phantom (Image Analysis, Lexington, KY) was placed under each participant and was visualized on each image obtained. The CT Hounsfield units (HU) from three areas of the liver were averaged to determine the average liver HU. We also measured the HU of the calibration phantom. We calculated liver phantom ratios (LPR) as the ratio between the average liver HU and the phantom HU as previously described [18]. Because the spleen was not visualized on all scans, the liver phantom ratio was used as the indexed standard. We defined NAFLD as a liver phantom ratio of ≤ 0.33, which was shown in our prior work to have a sensitivity of 70% and specificity of 98% for detecting NAFLD (based on a liver spleen ratio < 1.1 as the gold standard cut-off) [1].

Visceral adipose tissue (VAT) and subcutaneous adipose tissue (SAT) were measured using an image display window of -195 to -45 HU and a window center of -120 HU to identify pixels containing fat. A single reader manually traced the muscular abdominal wall separating the VAT and SAT compartments. VAT and SAT volumes were subsequently quantified using a semiautomatic segmentation technique at a dedicated offline workstation (Aquarius 3D Workstation; TeraRecon, San Mateo, CA) as described [17, 19].

### Covariates and baseline measurements

Covariate and baseline measurements were assessed at the second examination cycle (May 2008 to March 2011) for the Third Generation and Omni Cohort 2 participants and the ninth examination cycle (April 2011- March 2014) for the Offspring Cohort participants. Serum ALT, AST, high density lipoprotein (HDL), total cholesterol, triglycerides, glucose, platelets, and albumin levels were obtained from fasting morning samples using an automated Roche method (Roche cobas 501). Data on ethnicity was obtained from a self-administered questionnaire. Alcohol use and smoking status were assessed on the basis of physician-administrated questionnaires. Alcohol use was recorded as drinks per week or drinks per month. Participants were considered current smokers if they had smoked at least one cigarette per day in the year preceding the FHS examination. Using standard protocols, trained technicians measured heart rate, blood pressure, height, weight, and waist circumference in all participants as has been previously reported [17]. Body mass index (BMI) was defined as weight (kg)/height^2^ (m^2^). Diabetes was defined as a fasting plasma glucose ≥126 mg/dL or treatment with a hypoglycemic agent or insulin. Impaired fasting glucose (IFG) was defined as a fasting plasma glucose level of 100 to 125 mg/dL among those not treated for diabetes. Hypertension was defined as systolic blood pressure ≥140 mm Hg, diastolic blood pressure ≥90 mm Hg, or on treatment with an antihypertensive agent. Pulse pressure was calculated as the difference between systolic blood pressure and diastolic blood pressure. High triglycerides was defined by a measure of 150 mg/dL or higher. Low HDL cholesterol was defined as a HDL cholesterol < 50 mg/dL for women and < 40 mg/dL for men.

### Estimating risk of advanced fibrosis

In individuals with NAFLD (as defined by a LPR ≤ 0.33) (n = 575), serum markers of fibrosis were used to estimate the severity of fibrosis. These included the AST/ALT ratio, APRI, FIB4, and NFS. The AST/ALT ratio was calculated by dividing the serum AST by the ALT. We used the originally published cut-off of AST/ALT ratio ≤ 0.8 [11] and the more commonly used AST/ALT ratio ≤ 1 cut-off for excluding advanced fibrosis [7]. The APRI was calculated based on the published formula (APRI = [AST/upper limit of normal]/ platelet count [10^9^/L] x 100) [20]. We used the cut-offs of 0.5 for low and 1.5 for high probability of advanced fibrosis that have previously been published [20]. We calculated FIB4 by the formula: age(years) x AST[U/L]/(platelets [10^9^/L] x (ALT[U/L])^1/2^) [9]. For FIB4, we used the cut-offs of <1.30 for low, between 1.3–2.67 for indeterminate, and >2.67 for high probability of advanced fibrosis as previously published.[12] NFS was calculated according to the published formula (NFS = -1.675 + 0.037 x age [years] +0.094 x BMI [kg/m^2^] + 1.13 x impaired fasting glucose or diabetes [yes = 1, no = 0] + 0.99 x AST/ALT ratio– 0.013 x platelet [x 10^9^/L]– 0.66 x albumin g/dL) [10]. Participants were characterized into three categories based on the following, previously published cut-offs: NFS > 0.676 high probability advanced fibrosis, -1.455 ≤ NFS ≤ 0.676 indeterminate probability of advanced fibrosis, and NFS < -1.455 as low probability of advanced fibrosis [10].

### Statistical analysis

To describe the characteristics of the analysis population, we used means with standard deviations for continuous variables and percentages for categorical variables. Among participants with NAFLD on CT scan as defined by a LPR ≤ 0.33, we determined the percentage of participants with predicted fibrosis based on the AST/ALT ratio, APRI, FIB4, and the NFS. We hypothesized that approximately 10% of our sample would be at high risk for advanced fibrosis based on prior estimates in the population [21]. Since the NFS predicted a high risk for advanced fibrosis most similar to our *a priori* hypothesis, we continued the analysis using this model to categorized participants as low, indeterminate, or high risk for advanced fibrosis. Using multivariable linear and logistic regression models, we assessed the cross-sectional association between NAFLD with fibrosis according to NFS category (low risk fibrosis, indeterminate risk fibrosis, or high risk fibrosis) and various cardiometabolic risk factors including systolic blood pressure, diastolic blood pressure, pulse pressure, triglycerides, HDL cholesterol, hypertension, high triglycerides, and low HDL cholesterol. Model 1 adjusted for age, sex, smoking status, and drinks per day. For the analyses of systolic blood pressure, diastolic blood pressure and pulse pressure, model 1 was additionally adjusted for treatment for hypertension. For the analyses of triglycerides and HDL cholesterol, we also adjusted for treatment with lipid lowering medication in model 1. Because of the high correlation between NAFLD and obesity, we separately added BMI, a measure of general adiposity, and VAT, a measure of central adiposity, to the multivariable model in models 2 and 3, respectively.

## Results

### Study sample characteristics

The characteristics of the FHS sample (n = 1,968) are summarized in Table 1. 29.2% of the sample had NAFLD as defined as an LPR ≤ 0.33 on CT scan. Participants with NAFLD were slightly older, less likely to be women, and generally had a less favorable cardiovascular risk factor profile.

**Table 1:** Baseline characteristics of the study sample, by presence or absence of NAFLD.

	NAFLD (n = 575)	No NAFLD (n = 1393)	P value
Age (years)	61.2 (2.4)	60.8 (12.7)	0.48
Women (%)	42.3 (243)	55.0 (766)	< 0.001
Cohort			0.52
Offspring (%)	48.9 (281)	48.9 (681)	
Omni (%)	8.0 (46)	6.6 (92)	
Gen 3 (%)	43.1(248)	44.5 (620)	
Ethnicity			0.93
White (%)	95.6 (544)	95.3 (1320)	
Black (%)	1.4 (8)	1.6 (22)	
Asian (%)	1.8 (10)	2.1 (29)	
Native American (%)	0.0 (0)	0.1 (2)	
Multiracial (%)	1.2 (7)	0.9 (12)	
Smoking (%)	5.2 (30)	6.1 (85)	0.45
Alcohol (drinks/week)	2.7 (3.6)	2.7 (3.3)	0.81
Diabetes (%)	21.0 (121)	7.3 (102)	<0.001
IFG (%)	60.0 (345)	32.0 (446)	<0.001
Anti-hypertensive use (%)	50.3 (289)	33.2 (462)	<0.001
Lipid lowering therapy (%)	46.6 (268)	35.9 (500)	<0.001
BMI (kg/m^2^)	31.7(5.5)	27.3 (4.7)	<0.001
Obesity (BMI>30)	59.0 (339)	23.4 (326)	<0.001
Waist circumference (cm)	109.9 (16.7)	96.9 (13.2)	<0.001
Liver phantom ratio	0.27 (0.1)	0.37 (0.03)	<0.001
Systolic blood pressure (mmHg)	125.8 (14.3)	121.2 (15.5)	<0.001
Diastolic Blood Pressure (mmHg)	75.1 (9.8)	72.4 (8.9)	<0.001
Hypertension (%)	57.9 (333)	38.5 (536)	<0.001
Pulse pressure (mmHg)	50.7 (13.8)	48.8 (14.2)	0.007
Total Cholesterol (mg/dl)	181.8 (37.5)	187.4 (36.1)	0.002
HDL Cholesterol (mg/dl)	51.8 (14.7)	61.7 (17.7)	<0.001
Triglycerides (mg/dl)*	128.0 (95.0–176.0)	94.0 (71.0–126.0)	<0.001
ALT (IU/L)	29 (17)	22 (13)	<0.001
AST (IU/L)	24 (10)	22 (11)	<0.001
Albumin (g/dl)	4.4 (0.3)	4.4 (0.3)	0.27
Platelets (x10^9^/L)	241 (60)	239 (62)	0.59
Fasting Glucose (mg/dl)	108 (26)	98 (16)	<0.001
Metabolic Syndrome (%)	68.0 (391)	33.2 (462)	<0.001

IFG, impaired fasting glucose; BMI, body mass index; HDL, high density lipoprotein; ALT, alanine aminotransferase; AST, aspartate aminotransferase.

Continuous variables expressed as mean (sd), categorical variables as % (n).

* Values represent the median (interquartile range)

### Prediction of advanced fibrosis among participants with NAFLD according to AST/ALT ratio, APRI, and NFS

**Fig 1:**
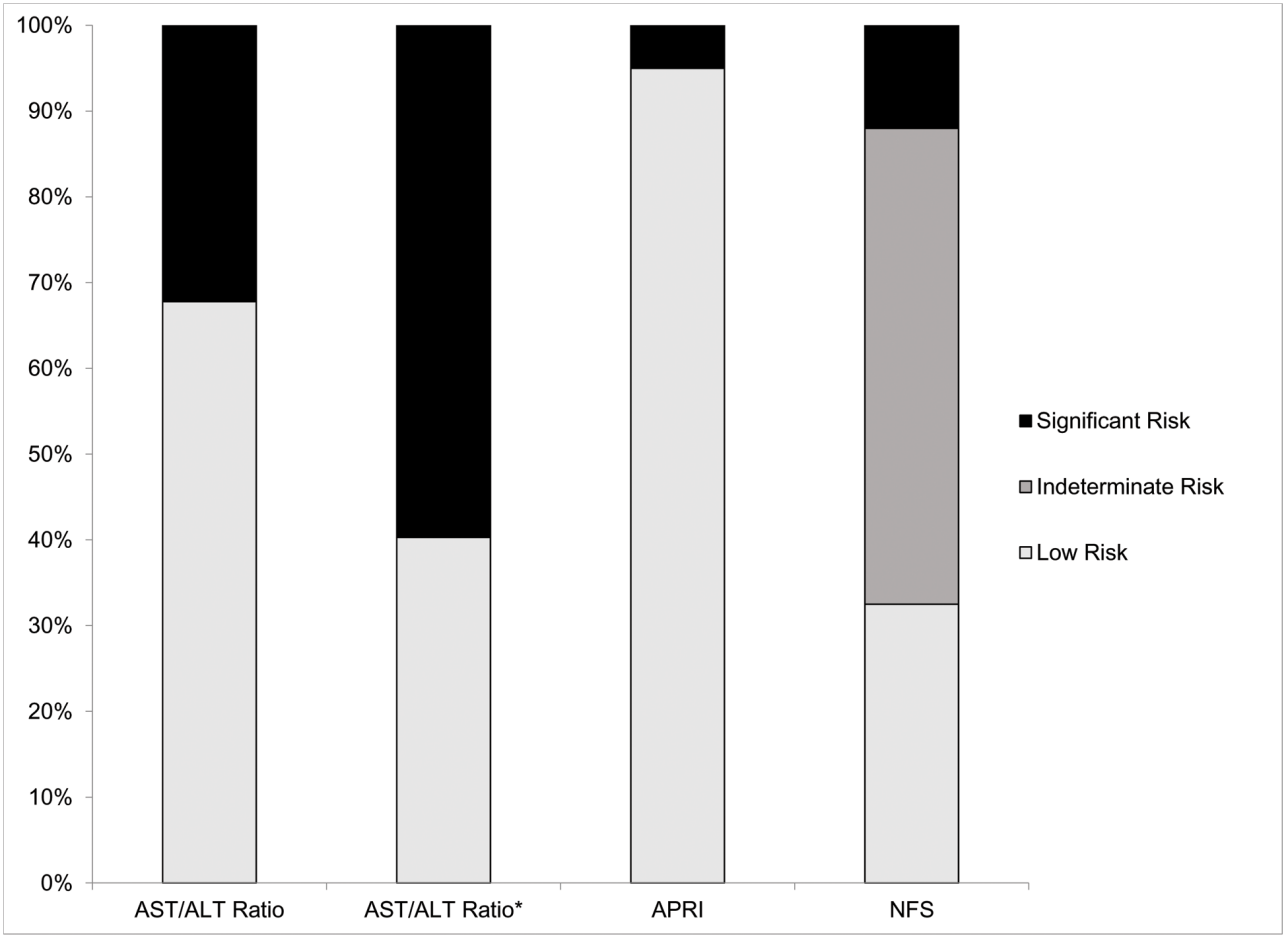
Prediction of the risk for liver fibrosis among participants with NAFLD (n = 575) according to the AST/ALT ratio, APRI, FIB4, and NFS. For the AST/ALT ratio, significant risk of fibrosis was defined as an AST/ALT ratio > 1.0 and absence of significant fibrosis as an AST/ALT ratio ≤ 1.0. *We also show the predicted risk of fibrosis for the AST/ALT ratio using the 0.8 cut-off. For the APRI, a significant risk/intermediate risk of fibrosis was defined as an APRI > 0.5 while an absence of fibrosis was defined as APRI ≤ 0.5. For the FIB4, we defined significant fibrosis as a FIB4 > 2.67 and a low risk of fibrosis as FIB4 < 1.30 with values in-between defined as indeterminate. For the NFS, we used the following definitions for the risk categories: high risk advanced fibrosis (NFS > 0.676), indeterminate risk for advanced fibrosis (1.455 ≤ NFS ≤ 0.676), and low risk for advanced fibrosis (NFS < -1.455).

For participants with NAFLD (n = 575), the percentage of participants with predicted fibrosis based on the AST/ALT ratio, APRI, FIB4, and NFS are summarized in Fig 1. Using the NFS, 12% of the sample (n = 69) had a high risk for advanced fibrosis (NFS > 0.676) while 33% of the sample (n = 187) had a low risk for advanced fibrosis (NFS < -1.455). The majority of participants (56%; n = 319) were at indeterminate risk of advanced fibrosis (-1.455 ≤ NFS ≤ 0.676). The APRI predicted a lower percentage of participants with significant/intermediate risk fibrosis (5%; n = 29). FIB4 preformed similarly to APRI with 4% (n = 23) of participants predicted to have significant fibrosis and an additional 35% (n = 201) of participants had an indeterminate level of fibrosis. The AST/ALT ratio at a cut off of 1.0 predicted a higher percentage of participants with significant risk of fibrosis (32%; n = 185).

### Characteristics of participants with NAFLD according to risk of fibrosis predicted by NFS

The characteristics of the participants with NAFLD (n = 575) according to risk of advanced fibrosis as predicted by the NFS is summarized in Table 2. As expected from the component variables of the NFS, advanced fibrosis is associated with older age (p<0.0001), higher BMI (p<0.0001), and increased prevalence of diabetes (p<0.0001) or impaired fasting glucose (p<0.0001). Of note, the mean ALT value for those with NAFLD and a high risk for advanced fibrosis was well below the normal threshold (ALT >19 U/L in women or >30 U/L in men).

**Table 2:** Characteristics of participants with fatty liver according to risk of fibrosis as predicted by the NAFLD Fibrosis Score.

	Low risk of advanced fibrosis (< -1.455) (n = 187)	Indeterminate risk of advanced fibrosis (-1.455–0.676) (n = 319)	High risk of advanced fibrosis (>0.676) (n = 69)
Age (years)	54.1 (10.4)	62.7 (11.3)	73.7 (9.9)
Women (%)	41.2 (77)	41.4 (132)	49.3 (34)
Cohort			
Offspring (%)	23.0 (43)	55.8 (178)	87.0 (60)
Omni (%)	10.7 (20)	7.5 (24)	2.9 (2)
Gen 3 (%)	66.3 (124)	36.7 (117)	10.1 (7)
Race			
White (%)	93.6 (175)	96.6 (308)	97.1 (67)
Black (%)	2.1 (4)	0.9 (3)	2.9 (2)
Asian (%)	2.7 (5)	1.9 (6)	0
Native American (%)	1.1 (2)	1.3 (4)	0
Smoking (%)	6.4 (12)	3.8 (12)	8.7 (6)
Alcohol (drinks/week)	2.8 (3.7)	2.8 (3.6)	1.9 (3.6)
Diabetes (%)	9.1 (17)	22.9 (73)	44.9 (31)
IFG (%)	29.4 (55)	69.9 (223)	97.1 (67)
Anti-hypertensive use (%)	36.9 (69)	51.1 (163)	82.6 (57)
Lipid lowering therapy (%)	34.8 (65)	49.8 (159)	63.8 (44)
BMI (kg/m^2^)	29.7 (4.4)	32.3 (5.3)	34.7 (6.8)
Obesity (BMI>30)	44.9 (84)	64.6 (206)	71.0 (49)
Waist circumference (cm)	103.4 (11.6)	111.2 (14.6)	121.2 (26.7)
Liver phantom ratio	0.27 (0.1)	0.26 (0.1)	0.29 (0.04)
Systolic blood pressure (mmHg)	123.0 (13.9)	126.1 (14.2)	131.8 (14.6)
Diastolic Blood Pressure (mmHg)	77.0 (9.8)	75.5 (8.9)	68.1 (11.2)
Hypertension (%)	45.5 (85)	58.9 (188)	87.0 (60)
Pulse pressure (mmHg)	46.0 (11.3)	50.6 (13.7)	63.7 (12.2)
Total Cholesterol (mg/dl)	192.1 (44.8)	178.5 (31.5)	169.4 (34.7)
HDL Cholesterol (mg/dl)	51.7 (14.3)	51.6 (14.9)	53.1 (14.8)
Triglycerides (mg/dl)*	120 (92–173)	134 (97–181)	126 (90–179)
ALT (IU/L)	34 (19)	29 (16)	18 (7)
AST (IU/L)	24 (8)	25 (11)	21 (7)
Albumin (g/dl)	4.5 (0.2)	4.4 (0.3)	4.2 (0.2)
Platelets (x10^9^/L)	282 (63)	226 (47)	197 (43)
Fasting Glucose (mg/dl)	101 (28)	110 (21)	121 (34)
Metabolic Syndrome (%)	48.1 (90)	74.0 (236)	94.2 (65)

IFG, impaired fasting glucose; BMI, body mass index; HDL, high density lipoprotein; ALT, alanine aminotransferase; AST, aspartate aminotransferase.

Continuous variables expressed as mean ± sd, categorical variables as %.

* Values represent the median (interquartile range).

### Multi-variable logistic regression models evaluating the association between risk of advanced fibrosis according to NFS and cardiometabolic risk factors

In multivariable models, participants with NAFLD and a high risk for advanced fibrosis had a lower diastolic blood pressure (Adjusted mean difference = -2.67 mm Hg; 95% CI -5.43–0.09 mm Hg; p = 0.06) and a wider pulse pressure (Adjusted mean difference = 6.87 mm Hg; 95% CI 3.33–10.42 mm Hg; p = 0.0002) compared to those with NAFLD and low risk of fibrosis Table 3. Results were strengthened after additional adjustment for BMI or VAT. There were no significant differences between those with NAFLD and a high risk of advanced fibrosis compared to those with NAFLD and a low risk of advanced fibrosis with respect to systolic blood pressure, HDL cholesterol or triglycerides. These results were similar when comparing those with NAFLD and high risk of fibrosis to the NAFLD and low or indeterminate risk fibrosis category (S1 Table).

**Table 3:** Multivariable linear regression models evaluating the association between NAFLD Fibrosis Score risk categories and continuous cardiometabolic risk factors.

	Low risk advanced fibrosis	Indeterminate risk advanced fibrosis		High risk advanced fibrosis	
	β estimates (95%CI)	β estimates (95%CI)	P-value	β estimates (95%CI)	P-value
Systolic blood pressure (mm Hg)					
MV*	Reference	0.72 (-1.93,3.37)	0.59	4.20 (-0.15,8.56)	0.06
MV + BMI	Reference	0.31 (-2.55,3.16)	0.83	3.38 (-1.48,8.23)	0.17
MV + VAT	Reference	0.34 (-2.38,3.06)	0.81	3.42 (-1.12,7.96)	0.14
Diastolic blood pressure (mm Hg)					
MV*	Reference	1.01 (-0.67,2.69)	0.24	-2.67 (-5.43,0.09)	0.06
MV + BMI	Reference	0.59 (-1.22,2.39)	0.52	-3.52 (-6.59,-0.45)	0.03
MV + VAT	Reference	0.64 (-1.07,2.36)	0.46	-3.42 (-6.29,-0.55)	0.02
Pulse pressure (mm Hg)					
MV*	Reference	-0.29 (-2.44,1.87)	0.79	6.87 (3.33,10.42)	0.0002
MV + BMI	Reference	-0.28 (-2.60,2.05)	0.82	6.90 (2.94,10.85)	0.0007
MV + VAT	Reference	-0.30 (-2.52,1.91)	0.79	6.85 (3.14,10.55)	0.0003
High Density Lipoprotein (mg/dL)					
MV*	Reference	-1.78 (-4.27,0.72)	0.16	-2.67 (-6.75,1.41)	0.20
MV + BMI	Reference	-0.98 (-3.67,1.70)	0.47	-1.04 (-5.61,3.52)	0.65
MV + VAT	Reference	-0.54 (-3.07,1.98)	0.67	-0.05 (-4.25,4.16)	0.98
Triglycerides (mg/dL)					
MV*	Reference	9.24 (-6.35,24.83)	0.25	13.81 (-11.68,39.30)	0.29
MV + BMI	Reference	5.81 (-10.98,22.60)	0.50	6.75 (-21.80,35.29)	0.64
MV + VAT	Reference	4.29 (-11.64,20.22)	0.60	3.28 (-23.23,29.79)	0.81

NAFLD, Non-alcoholic fatty liver disease; BMI, body mass index; VAT, visceral adipose tissue.

*Multivariate model (MV): Covariate adjustment included age, sex, smoking status (current vs no), and drinks per day. For the analyses with systolic blood pressure and diastolic blood pressure, the MV model also included adjustment for treatment for hypertension. For the analyses with high density lipoprotein and triglycerides, the MV model also included adjustment for treatment with lipid lowering medication.

Individuals with NAFLD and a high risk for advanced fibrosis had a higher adjusted odds of hypertension (OR 2.92 95% CI 1.35–6.34; p = 0.007) compared to participants with NAFLD and a low or indeterminate risk for advanced fibrosis (S2 Table). These results were slightly attenuated when BMI (OR = 2.33; 95% CI 1.05–5.19; p = 0.04) or VAT (OR = 2.39; 95% CI 1.09–5.24; p = 0.03) was added to the multivariable model; however, the results remained significant. When comparing to NAFLD participants with a low risk of advanced fibrosis, those with NAFLD and a high risk of advanced fibrosis had a 3.06 increased odds of hypertension (95% CI 1.33–7.04; p = 0.009); however, this was no longer statistically significant when BMI (OR = 2.05; 95% CI 0.83–5.07; p = 0.12) or VAT (OR = 2.19; 95% CI 0.93–5.18; p = 0.07) was added to the multivariable model (Table 4). There were no differences between the odds of low HDL cholesterol or high triglycerides for those with NAFLD and a high risk of advanced fibrosis compared to those with low risk and low or indeterminate risk of advanced fibrosis.

**Table 4:** Multivariable logistic regression models evaluating the association between NAFLD Fibrosis Score risk categories and dichotomous cardiometabolic risk factors.

	Low risk advanced fibrosis	Indeterminate risk advanced fibrosis		High risk advanced fibrosis	
	OR (95%CI)	OR (95%CI)	P-value	OR (95%CI)	P-value
Hypertension (yes vs no)					
MV*	Reference	1.06 (0.71,1.59)	0.77	3.06 (1.33,7.04)	0.009
MV + BMI	Reference	0.87 (0.56,1.36)	0.55	2.05 (0.83,5.07)	0.12
MV + VAT	Reference	0.90 (0.59,1.37)	0.62	2.19 (0.93,5.18)	0.07
Low HDL cholesterol (yes vs no)					
MV*	Reference	1.27 (0.84,1.93)	0.26	1.45 (0.72,2.89)	0.30
MV + BMI	Reference	1.23 (0.78,1.93)	0.37	1.35 (0.62,2.94)	0.45
MV + VAT	Reference	1.14 (0.74,1.75)	0.55	1.15 (0.55,2.38)	0.71
High Triglycerides (yes vs no)					
MV*	Reference	1.46 (0.98,2.18)	0.06	2.03 (0.99,4.17)	0.05
MV + BMI	Reference	1.22 (0.79,1.89)	0.36	1.41 (0.64,3.12)	0.39
MV + VAT	Reference	1.25 (0.82,1.88)	0.30	1.46 (0.69,3.08)	0.32

NAFLD, Non-alcoholic fatty liver disease; OR, odds ratio; HDL, high density lipoprotein; BMI, body mass index; VAT, visceral adipose tissue.

*Multivariate model (MV): Covariate adjustment included age, sex, smoking status (current vs no), and drinks per day.

## Discussion

In this unselected community based cohort study, our findings are threefold. First, when applied to a community-based sample with radiographically defined NAFLD, the AST/ALT ratio, APRI, FIB4, and NFS give widely disparate predictions of the risk for significant hepatic fibrosis. Second, in adjusted models, participants with NAFLD and a high risk of advanced fibrosis as predicted by the NFS had evidence of worsened vascular function as evidenced by a lower diastolic blood pressure, wider pulse pressure, and increased odds of hypertension compared to participants with NAFLD and a low or indeterminate risk for advanced fibrosis. Finally, although the prevalence of lipid disorders was high among those with NAFLD and a high risk for advanced fibrosis, in adjusted models, there were no significant differences in triglycerides, HDL cholesterol or the odds of high triglycerides or low HDL cholesterol between those at high risk of fibrosis compared to those at low and low or indeterminate risk of fibrosis.

We advance the current literature by demonstrating the variability in predictions of significant fibrosis by current non-invasive fibrosis markers in an unselected, community-based sample. Previously there have been only a limited number of community- or population-based studies which have used non-invasive fibrosis markers in participants with NAFLD. A study utilizing the third National Health and Nutrition Examination Survey (NHANES III) population and another study in a community-based sample both did not report the percentage of participants with NAFLD and significant fibrosis according to the various fibrosis markers examined.[14, 22] A study in a cohort of adults with type 2 diabetes and ultrasound-defined NAFLD found poor agreement on the presence of advanced fibrosis between 4 serum based biomarkers of fibrosis, including AST/ALT ratio, APRI, FIB4, and the European Liver Fibrosis panel, and measures of liver fibrosis using transient elastography.[23] In the subgroup of participants with NAFLD in this study, the percentage of participants with probable liver fibrosis ranged from 0.4% using the APRI, 16.7% using the AST/ALT ratio, to as high as 63.8% using the FIB4. The findings in our study extend this prior work by demonstrating the variability in 4 validated-fibrosis markers in a community-based sample not pre-selected for diabetes or other metabolic diseases. Together these studies demonstrate the need for caution when applying the currently available models that predict fibrosis in populations with a low pre-test probability of fibrosis. In our study, we found the NFS predicted 12% of the participants with NAFLD were at high risk for advanced fibrosis which is similar to estimates in other low risk cohorts.[21] Since liver biopsy is not ethical or practical to implement on a large scale and traditional imaging modalities do not diagnosis NASH or mild-to-moderate fibrosis, there is a need for the development of non-invasive fibrosis markers in a wide variety of populations, including community-based cohorts.

When using the NFS to define risk for advanced fibrosis, participants with NAFLD and a high risk for advanced fibrosis had worsened arterial stiffness, as indicated by a wider pulse pressure [24], and an increased odds of hypertension compared to those with NAFLD and a low or indeterminate risk of advanced fibrosis. Arterial stiffness is increasingly being recognized as an important prognostic indicator and potential therapeutic target in patients with hypertension.[25] It is well known that elevated pulse pressure predisposes to myocardial infarction, atrial fibrillation, and congestive heart failure, independent of elevations to systolic blood pressure.[26–28] In community-based cohorts, ultrasound defined NAFLD has been associated with higher measures of arterial stiffness including the brachial-ankle pulse wave velocity and the carotid-femoral pulse wave velocity.[29–31] Future studies are needed to determine the role of arterial stiffness in the pathogenesis of NAFLD and if interventions targeted to improve arterial stiffness are of benefit to patients with NAFLD.

It is well established that NAFLD is associated with an increased risk of elevated total cholesterol, triglycerides, and low-density lipoprotein (LDL) cholesterol and lower HDL cholesterol.[1] This finding was confirmed in our study as we demonstrated a higher prevalence of lipid disorders among those with NAFLD compared to participants without NAFLD. However, we found no differences in triglycerides or HDL cholesterol between those with NAFLD and a high risk for advanced fibrosis compared to those at a low or indeterminate risk for fibrosis in adjusted models. One possible explanation for these findings is that we are observing a treatment effect since over 60% of participants with NAFLD and a high risk for advanced fibrosis were on treatment with a lipid lowering medication in our sample. However, the most common lipid lowering medications exert small effects on lowering triglycerides and have little effect on raising HDL cholesterol. For this reason, we suspect that treatment for hyperlipidemia does not fully explain the lack of significant differences in triglyceride and HDL cholesterol levels between fibrosis risk categories in our sample. Given our relatively low sample size of participants with a high risk for advanced fibrosis, it is possible that we lack adequate power to detect a difference between the fibrosis risk categories. Alternatively, it may be that differences in HDL cholesterol and triglycerides may not contribute much to the worsened cardiovascular disease endpoints observed in patients with NAFLD and a high risk for advanced fibrosis. Future studies with larger sample sizes should continue to explore the cardiometabolic risk factors that may contribute to an increased risk for cardiovascular disease in patients with NAFLD and fibrosis.

The major strengths of our investigation include the assessment of multiple models for predicting liver fibrosis in the context of a large community-based sample that has not been selected for NAFLD. The FHS is a well-characterized sample with a thorough assessment of both covariates and outcomes using standardized measurements. We are able to add to the current literature by adjusting for several important confounders in exploring the association between NAFLD with low, indeterminate, or high risk for advanced fibrosis with multiple cardiovascular risk factors.

Our investigation has a number of limitations which are important to discuss. First, our study utilizes a cross-sectional design so we are unable to make any inferences on causality or temporality. Also, the FHS is largely white, which may impact the generalizability to individuals of non-European ancestry. Additionally, by defining NAFLD based on CT imaging, which is insensitive to mild liver fat, we are likely underrepresenting the burden of NAFLD in the population. Since hepatic steatosis can diminish as liver fibrosis progresses, it is possible that participants with more severe fibrosis were excluded from the analysis. We also lack information about viral hepatitis status and other chronic liver conditions which can cause the appearance of liver fat on CT scan which may have biased our findings towards the null. Finally, we utilized published cut-offs for each of the serum based fibrosis panels evaluated; however, these cut-offs were defined in hospital-based cohorts or case-control studies and have not been validated in the general population. It is likely that the choice of the cut-off point influenced our results.

### Conclusion

In a community based cohort of participants with evidence of NAFLD based on CT imaging, the AST/ALT ratio, APRI, FIB4, and NFS predicted a widely disparate risk for liver fibrosis, indicating the need to use caution when using these markers, particularly in cohorts with low disease prevalence. Participants with NAFLD and a high risk for advanced fibrosis as predicted by the NFS had a wider pulse pressure and an increased odds of hypertension. Whether modifying these risk factors impacts cardiovascular endpoints in NAFLD patients remains unknown and should be explored in future studies.

## Supporting Information

S1 Table Multivariable linear regression models evaluating the association between high risk of advanced fibrosis based on NAFLD Fibrosis Score and continuous cardiometabolic risk factors compared to those at low or indeterminate risk of advanced fibrosis. (DOCX)

S2 Table Multivariable logistic regression models evaluating the association between high risk of advanced fibrosis based on NAFLD Fibrosis Score and dichotomous cardiometabolic risk factors compared to those at low or indeterminate risk of advanced fibrosis. (DOCX)
