# Supplementary material for: The Association between Non-Invasive Hepatic Fibrosis Markers and Cardiometabolic Risk Factors in the Framingham Heart Study
Source: PLoS One. 2016 Jun 24;11(6):e0157517. doi: 10.1371/journal.pone.0157517 (PMC4920364; doi:10.1371/journal.pone.0157517)
Supplement: S1 Table — (DOCX) [file pone.0157517.s001.docx]

**S1 Table: Multivariable linear regression models evaluating the association between high risk of advanced fibrosis based on NAFLD Fibrosis Score and continuous cardiometabolic risk factors compared to those at low or indeterminate risk of advanced fibrosis.**

|  | Low/Indeterminate risk advanced fibrosis | High risk advanced fibrosis** | |
| --- | --- | --- | --- |
|  | β estimates (95%CI) | β estimates (95%CI) | P-value |
| Systolic blood pressure (mm Hg) |  |  |  |
| MV* | Reference | 3.61 (-0.16,7.39) | 0.06 |
| MV + BMI | Reference | 3.42 (-1.12,7.96) | 0.14 |
| MV + VAT | Reference | 3.12 (-0.73,6.97) | 0.11 |
| Diastolic blood pressure (mm Hg) |  |  |  |
| MV* | Reference | -3.50 (-5.89,-1.10) | 0.01 |
| MV + BMI | Reference | -4.10 (-6.60,-1.60) | 0.001 |
| MV + VAT | Reference | -3.99 (-6.43,-1.56) | 0.001 |
| Pulse pressure (mm Hg) |  |  |  |
| MV* | Reference | 7.11 (4.04,10.18) | <0.0001 |
| MV + BMI | Reference | 7.17 (3.95,10.39) | <0.0001 |
| MV + VAT | Reference | 7.11 (3.98,10.25) | <0.0001 |
| High Density Lipoprotein (mg/dL) |  |  |  |
| MV* | Reference | -1.22 (-4.76,2.32) | 0.50 |
| MV + BMI | Reference | -0.07 (-3.78,3.64) | 0.97 |
| MV + VAT | Reference | 0.43 (-3.13,3.99) | 0.81 |
| Triglycerides (mg/dL) |  |  |  |
| MV* | Reference | 6.27 (-15.83,28.36) | 0.58 |
| MV + BMI | Reference | 1.01 (-22.21,24.22) | 0.93 |
| MV + VAT | Reference | -0.52 (-22.96,21.92) | 0.96 |

NAFLD, Non-alcoholic fatty liver disease; BMI, body mass index; VAT, visceral adipose tissue.

*Multivariate model (MV): Covariate adjustment included age, sex, smoking status (current vs no), and drinks per day. For the analyses with systolic blood pressure and diastolic blood pressure, the MV model also included adjustment for treatment for hypertension. For the analyses with high density lipoprotein and triglycerides, the MV model also included adjustment for treatment with lipid lowering medication.
