# Supplementary material for: The Association between Non-Invasive Hepatic Fibrosis Markers and Cardiometabolic Risk Factors in the Framingham Heart Study
Source: PLoS One. 2016 Jun 24;11(6):e0157517. doi: 10.1371/journal.pone.0157517 (PMC4920364; doi:10.1371/journal.pone.0157517)
Supplement: S2 Table — (DOCX) [file pone.0157517.s002.docx]

**S2 Table: Multivariable logistic regression models evaluating the association between high risk of advanced fibrosis based on NAFLD Fibrosis Score and dichotomous cardiometabolic risk factors compared to those at low or indeterminate risk of advanced fibrosis.**

|  | Low/Indeterminate risk advanced fibrosis | High risk advanced fibrosis | |
| --- | --- | --- | --- |
|  | OR (95%CI) | OR (95%CI) | P-value |
| Hypertension (yes vs no) |  |  |  |
| MV* | Reference | 2.92 (1.35,6.34) | 0.007 |
| MV + BMI | Reference | 2.33 (1.05,5.19) | 0.04 |
| MV + VAT | Reference | 2.39 (1.09,5.24) | 0.03 |
| Low HDL cholesterol (yes vs no) |  |  |  |
| MV* | Reference | 1.19 (0.65,2.18) | 0.57 |
| MV + BMI | Reference | 1.10 (0.58,2.08) | 0.77 |
| MV + VAT | Reference | 1.02 (0.55,1.90) | 0.95 |
| High Triglycerides (yes vs no) |  |  |  |
| MV* | Reference | 1.51 (0.79,2.87) | 0.21 |
| MV + BMI | Reference | 1.16 (0.59,2.28) | 0.66 |
| MV + VAT | Reference | 1.21 (0.63,2.35) | 0.57 |

NAFLD, Non-alcoholic fatty liver disease; OR, odds ratio; HDL, high density lipoprotein; BMI, body mass index; VAT, visceral adipose tissue.

*Multivariate model (MV): Covariate adjustment included age, sex, smoking status (current vs no), and drinks per day.
